# Risk factors for physical disability in patients with leprosy disease in Yunnan, China: Evidence from a retrospective observational study

**DOI:** 10.1371/journal.pntd.0009923

**Published:** 2021-11-10

**Authors:** Xiaohua Chen, Hong-bing Liu, Tie-Jun Shui, Shun Zha

**Affiliations:** 1 Beijing Tropical Medicine Research Institute, Beijing Friendship Hospital, Capital Medical University, Beijing, China; 2 Beijing Key Laboratory for Research on Prevention and Treatment of Tropical Diseases, Capital Medical University, Beijing, China; 3 Lincang City Center for Disease Control and Prevention, Lincang, Yunnan, China; 4 Yunnan Center for Disease Control and Prevention, Yunnan, China; Adolfo Lutz Institute of Sao Jose do Rio Preto, BRAZIL

## Abstract

**Background:**

Leprosy is potentially debilitating. The risk factors related to physical disabilities associated with leprosy disease in Yunnan, China was not clear.

**Methodology/Principal findings:**

We studied 10644 newly detected leprosy patients from Yunnan, China, from 1990 to 2019. Factors associated with Grade 1 (G1D) and Grade 2 (G2D) physical disabilities or overall physical disabilities (combined G1D and G2D) associated with leprosy were analyzed using multinomial and ordinal logistic regression analyses. The following factors were associated with the development of physical disability in these patients with leprosy: delayed diagnosis [odds ratio (OR): 5.652, 4.399, and 2.275; 95% confidence intervals (CIs): 4.516–7.073, 3.714–5.212, and 2.063–2.509; for ≥ 10, 5–10 y, and 2–5 years, respectively], nerve damage (OR: 3.474 and 2.428; 95% CI: 2.843–4.244, and 1.959–3.008; for 2 and 1 damaged nerves, respectively), WHO classification of PB (OR: 1.759; 95% CI: 1.341–2.307), Ridley-Jopling classification (OR: 1.479, 1.438, 1.522 and 1.239; 95% CI: 1.052–2.079, 1.075–1.923, 1.261–1.838, and 1.072–1.431; for TT, BT, BB, and BL when compared with LL, respectively), advanced age (OR: 1.472 and 2.053; 95% CI: 1.106–1.960 and 1.498–2.814; for 15–59 and over 60 years old, respectively), zero skin lesions (OR: 1.916; 95% CI: 1.522–2.413), leprosy reaction (OR: 1.528; 95% CI: 1.195–1.952), rural occupation (OR: 1.364; 95% CI: 1.128–1.650), Han ethnicity (OR: 1.268; 95% CI: 1.159–1.386), and male sex (OR: 1.128; 95% CI: 1.024–1.243).

**Conclusions:**

Delayed diagnosis, nerve damage, no skin lesions, WHO and Ridley-Jopling classifications, leprosy reactions, advanced age, rural occupation, Han ethnicity, and male sex were associated with disability in leprosy patients. Identifying risk factors could help to prevent physical disability.

## Author summary

Leprosy, caused by *Mycobacterium leprae* (*M*. *leprae*), is a potentially disabling infectious disease. After achieving the national elimination target, the focus of leprosy care is now shifting from implementing multidrug therapy (MDT) to accelerating the reduction in disease burden, especially stopping leprosy and its complications, with targets for 2020 of 0 new child cases with Grade 2 disability (G2D) and <1 per million population new leprosy cases with G2D. We assessed physical disabilities and deformities of newly detected leprosy patients in Yunnan, an endemic area of leprosy disease in China, from 1990–2019 and the clinical and demographic factors associated with physical disabilities and deformities. We found that delayed diagnosis, nerve damage, WHO and Ridley-Jopling classifications, lack of skin lesions, leprosy reactions, advanced age, rural occupation, Han ethnicity, and male sex were risk factors for physical disabilities in patients with leprosy disease. The findings are important because physical disability is the main sequelae of leprosy disease that leads to stigma, discrimination, and socioeconomic burden. Understanding the related risk factors would be helpful for preventing the physical disability associated with leprosy disease.

## Introduction

Leprosy, caused by *Mycobacterium leprae* (*M*. *leprae*), is a potentially disabling infectious disease. When *M*. *leprae* settles into skin tissues and human peripheral nerves, the development of an immunologic response is induced. The action of the bacillus in the body and the inflammatory process lead to neuritis, which compromises autonomous, sensory and/or motor neural function. When neuritis impacts individuals who do not receive proper treatment, the process may become chronic, thus leading to the characteristic physical disabilities of the disease [1].

In 2019, 10813 leprosy cases with Grade 2 disabilities at diagnosis (G2D) were reported globally, and the proportion of G2D cases was 5.3% among new cases, corresponding to 1.2 per million population [2]. However, disability is unevenly distributed, with a relatively high rate of G2D continuously reported in recent years in India [3], Brazil [4,5], and China [6]. In India, 5245 leprosy cases with G2D were identified in 2016, corresponding to 2.9 per million population [3]. In Brazil, the proportion of new leprosy cases with G2D was 7.9%, and the rate was 8.42 per 1 million population in 2016 [4]. From 2012 to 2016, the mean rate of leprosy new case detection with G2D in Brazil was 10.5 per 1 million inhabitants [5]. In China, a relatively high level of G2D has been continuously reported in recent years. The percentage of G2D in newly detected cases was 19.0% in 2018, similar to the data in 2017 [6]. In the absence of verifiable data, it is estimated that 3–4 million people are living with visible impairments or deformities due to leprosy [7].

Due to the large number of people with disability/deformity due to leprosy, the World Health Organization (WHO) launched a 5-year global leprosy strategy in 2016 to reduce the disease burden and the prevalence of deformities [8]. Disabilities/deformities can lead to problems such as decreased ability to work, limited social life, and psychological problems, and they are responsible for stigma and prejudice against individuals with the disease [9,10]. Therefore, monitoring the prevalence and evaluating the risk factors for disabilities associated with leprosy are needed.

Yunnan Province is located in southwestern China and has the highest burden of leprosy in China. In the past 30 years (from 1990–2019), 11052 newly diagnosed leprosy patients were registered in the Leprosy Management Information System (LEPMIS), and the percentage of those with a disability at diagnosis ranged from 10.34% to 32.02% for G2D [11]. These numbers call attention to the need to perform studies evaluating epidemiological and clinical prevalence associated with physical impairment. This study aims to assess the characteristics and risk factors associated with physical disability and deformity in leprosy patients based on newly diagnosed leprosy patients in Yunnan, China.

## Methods

### Ethics statement

The study design and data analysis were approved by the Ethics Committee of the Yunnan CDC, Yunnan, China. Individual identifying information was not available and therefore not used.

### Data sources

The data for this retrospective observational study were collected from the LEPMIS in China. We systematically screened the case data of patients with leprosy from local hospitals and the Center of Disease Control and Prevention of Yunnan Province (CDC) in Yunnan, China. The inclusion criterion was newly detected leprosy cases from 1990–2019 in Yunnan, China. The diagnosis of leprosy by clinicians met the diagnostic criteria issued by the Ministry of Health of the People’s Republic of China [12]. The exclusion criteria were relapsed, imported, or revisited leprosy cases and no information or unclear information on the disability status of the leprosy patients. The newly detected leprosy patients were diagnosed with no disability (G0D), Grade 1 disability (G1D) or Grade 2 disability (G2D) and formed the sample for this study.

### Classification of disabilities and nerve involvement

All the patients included in this study with a confirmed diagnosis of leprosy disease were evaluated for physical impairment and nerve involvement based on the objective scale of physical impairment as defined by the WHO [13]. The classification related to physical impairment was as follows: Grade 0: no impairment, Grade 1: loss of sensation, and Grade 2: visible impairment.

### Variables

The demographics and clinical data were collected for this study. The patients’ basic demographic information included sex, date of birth, ethnicity, and occupation. The clinical characteristics included the age of diagnosis, date of symptom onset, date of diagnosis, detection mode, skin lesion, nerve damage, contact history, leprosy reaction, classification of disability (G0D, G1D, and G2D), Ridley-Jopling (clinical) classification, and WHO operational classification. Diagnosis duration was defined as the time duration between the onset of symptoms and the confirmed diagnosis.

### Statistical analysis

The Kolmogorov–Smirnov test was used to test the distribution of the patient characteristics. The results of descriptive analyses are presented as the mean ± standard deviation (SD), min to max, and medians and interquartile ranges (IQRs) for continuous variables and as counts and percentages in each category for categorical variables. Chi-square tests were used to examine differences in proportions of categorical variables between different groups. To explore the risk factors associated with G1D and G2D separately and the overall risk of physical disability (combined G1D and G2D), multinomial and ordinal logistic regression models were used to estimate the odds ratios (ORs) and 95% confidence intervals (CIs). All analyses were performed using SPSS, version 16.0 (SPSS Inc). P values ≤0.05 were considered significant.

## Results

### Characteristics of leprosy patients with physical disabilities

Table 1 shows the general characteristics of the study population. During the thirty-year study period from 1990 to 2019, 11616 records of leprosy cases in Yunnan, China, were retrieved, and 564 records were excluded as relapsed, imported, or revisited cases. A total of 11052 newly diagnosed leprosy cases were identified, and 96.3% of patients (n = 10644) had been assessed regarding the level of physical disability at the time of diagnosis. Of the 10644 patients with leprosy disease, 7242 (68.04%), 1165 (10.59%) and 2237 (21.02%) were categorized as G0D, G1D and G2D, respectively (Table 1). The median age was 35.00 years, and 70.46% were men. Among all patients, 47.41% were of Han ethnicity, and 91.74% were farmers. Overall, 64.22% of patients were diagnosed in the early stage, 64.41% were detected by passive case detection, 69.02% had a contact history with leprosy patients and 2.8% had leprosy reactions. Regarding the operational classification, 66.69% were multibacillary and 33.31% were paucibacillary (Table 1). Regarding the Ridley and Jopling classification, 40.50% were borderline-lepromatous (BL), 23.95% were borderline-tuberculoid (BT), 13.25% were lepromatous (LL), 10.89% were tuberculoid (TT), 9.82% were borderline-borderline (BB), and 1.53% were indeterminate (I) forms of leprosy (Table 1). The median diagnosis durations were 13, 16, and 29 months for leprosy patients with G0D, G1D, and G2D, respectively.

**Table 1:** Characteristics of New detected Leprosy Cases in Yunnan, China from 1990–2019, Grouped by Physical Disablity Grade.

		Grade 0		Grade 1		Grade 2		Physical disability		Total		P*
Characteristics		(n =)	%	(n =)	%	(n =)	%	(n =)	%	(n =)	%	
**Patient Demographic Characteristics**												
**Total**		7242	68.04%	1165	10.95%	2237	21.02%	3402	31.96%	10644	100.00%	
**Gender, No.(%)**	Male	5055	69.80%	811	69.79%	1634	73.04%	2445	71.87%	7500	70.46%	0.000
	Female	2187	30.20%	354	30.46%	603	26.96%	957	28.13%	3144	29.54%	
**Age,y**	**Median (IQR)**	33	(25–45)	35	(27–47)	40.5	(29–54)	39	(29–52)	35	(26–47)	
	Mean±SD (Min-Max)	35.32±15.16	(1–95)	37.74±14.67	(1–87)	42.15±15.64	(6–96)	40.64±15.45	(1–96)	36.99±15.04	(1–96)	
**Age Group,y**	0–14	373	5.15%	34	2.92%	32	1.43%	66	1.94%	439	4.12%	0.000
	15–19	537	7.42%	74	6.35%	111	4.96%	185	5.44%	722	6.78%	
	20–29	1897	26.19%	251	21.55%	442	19.76%	693	20.37%	2590	24.33%	
	30–39	1898	26.21%	342	29.36%	496	22.17%	838	24.63%	2736	25.70%	
	40–49	1249	17.25%	213	18.28%	446	19.94%	659	19.37%	1908	17.93%	
	50–59	774	10.69%	145	12.45%	372	16.63%	517	15.20%	1291	12.13%	
	60–69	380	5.25%	70	6.01%	231	10.33%	301	8.85%	681	6.40%	
	70–79	120	1.66%	32	2.75%	90	4.02%	122	3.59%	242	2.27%	
	80**~**	14	0.19%	4	0.34%	17	0.76%	21	0.62%	35	0.33%	
**Ethinic Group**	Han	3261	45.03%	635	54.51%	1150	51.41%	1785	52.47%	5046	47.41%	0.000
	Yi	937	12.94%	191	16.39%	267	11.94%	458	13.46%	1395	13.11%	
	Zhuang	755	10.43%	82	7.04%	173	7.73%	255	7.50%	1010	9.49%	
	Miao	699	9.65%	125	10.73%	228	10.19%	353	10.38%	1052	9.88%	
	Dai	520	7.18%	31	2.66%	73	3.26%	104	3.06%	624	5.86%	
	Missing data	440	6.08%	39	3.35%	134	5.99%	173	5.09%	613	5.76%	
	Lahu	175	2.42%	12	1.03%	56	2.50%	68	2.00%	243	2.28%	
	Bai	130	1.80%	11	0.94%	52	2.32%	63	1.85%	193	1.81%	
	Hani	88	1.22%	8	0.69%	30	1.34%	38	1.12%	126	1.18%	
	Tibetan	78	1.08%	9	0.77%	23	1.03%	32	0.94%	110	1.03%	
	Lisu	36	0.50%	6	0.52%	13	0.58%	19	0.56%	55	0.52%	
	Yao	33	0.46%	0	0.00%	6	0.27%	6	0.18%	39	0.37%	
	Hui	23	0.32%	10	0.86%	6	0.27%	16	0.47%	39	0.37%	
	Wa	19	0.26%	3	0.26%	10	0.45%	13	0.38%	32	0.30%	
	Jinuo	10	0.14%	1	0.09%	3	0.13%	4	0.12%	14	0.13%	
	Naxi	10	0.14%	0	0.00%	1	0.04%	1	0.03%	11	0.10%	
	Buyi	5	0.07%	0	0.00%	0	0.00%	0	0.00%	5	0.05%	
	Bulang	4	0.06%	0	0.00%	3	0.13%	3	0.09%	7	0.07%	
	Jingpo	4	0.06%	1	0.09%	3	0.13%	4	0.12%	8	0.08%	
	Achang	4	0.06%	0	0.00%	2	0.09%	2	0.06%	6	0.06%	
	Uighur	2	0.03%	0	0.00%	0	0.00%	0	0.00%	2	0.02%	
	Pumi	1	0.01%	0	0.00%	2	0.09%	2	0.06%	3	0.03%	
	Du	1	0.01%	1	0.09%	1	0.04%	2	0.06%	3	0.03%	
	Li	1	0.01%	0	0.00%	1	0.04%	1	0.03%	2	0.02%	
	De’Ang	1	0.01%	0	0.00%	0	0.00%	0	0.00%	1	0.01%	
	Nu	1	0.01%	0	0.00%	0	0.00%	0	0.00%	1	0.01%	
	Tujia	1	0.01%	0	0.00%	0	0.00%	0	0.00%	1	0.01%	
	Dong	1	0.01%	0	0.00%	0	0.00%	0	0.00%	1	0.01%	
	Manchu	1	0.01%	0	0.00%	0	0.00%	0	0.00%	1	0.01%	
	Mongol	1	0.01%	0	0.00%	0	0.00%	0	0.00%	1	0.01%	
**Occupation**	**Rural**	6676	92.18%	1100	94.42%	2152	96.20%	3252	95.59%	9928	93.27%	0.000
	Farmer	6567	90.68%	1081	92.79%	2117	94.64%	3198	94.00%	9765	91.74%	
	Others	79	1.09%	10	0.86%	26	1.16%	36	1.06%	115	1.08%	
	Farmer labourer	24	0.33%	8	0.69%	7	0.31%	15	0.44%	39	0.37%	
	Herdsman	4	0.06%	0	0.00%	1	0.04%	1	0.03%	5	0.05%	
	Seafarers and long-distance drivers	2	0.03%	1	0.09%	1	0.04%	2	0.06%	4	0.04%	
	Fisherman	0	0.00%	0	0.00%	0	0.00%	0	0.00%	0	0.00%	
	**Urban**	566	7.82%	65	5.58%	85	3.80%	150	4.41%	716	6.73%	
	Student	302	4.17%	26	2.23%	25	1.12%	51	1.50%	353	3.32%	
	Worker	97	1.34%	18	1.55%	30	1.34%	48	1.41%	145	1.36%	
	Children	49	0.68%	9	0.77%	4	0.18%	13	0.38%	62	0.58%	
	Clerks	48	0.66%	1	0.09%	7	0.31%	8	0.24%	56	0.53%	
	Housework and unemployment	30	0.41%	4	0.34%	6	0.27%	10	0.29%	40	0.38%	
	Self employed	11	0.15%	2	0.17%	0	0.00%	2	0.06%	13	0.12%	
	Retired	10	0.14%	1	0.09%	5	0.22%	6	0.18%	16	0.15%	
	Business Services	10	0.14%	0	0.00%	2	0.09%	2	0.06%	12	0.11%	
	Teacher	4	0.06%	3	0.26%	6	0.27%	9	0.26%	13	0.12%	
	Nursery child	2	0.03%	0	0.00%	0	0.00%	0	0.00%	2	0.02%	
	Public place attendant	2	0.03%	0	0.00%	0	0.00%	0	0.00%	2	0.02%	
	Technical personnel	1	0.01%	0	0.00%	0	0.00%	0	0.00%	1	0.01%	
	Food and beverage industry	0	0.00%	1	0.09%	0	0.00%	1	0.03%	1	0.01%	
	Babysitters and Nannies	0	0.00%	0	0.00%	0	0.00%	0	0.00%	0	0.00%	
	Medical staff	0	0.00%	0	0.00%	0	0.00%	0	0.00%	0	0.00%	
**Patient Clinic Characteristics**												
**Duration**												
**Delay Diagnosis, y**	< 2	5216	72.02%	735	63.09%	885	39.56%	1620	47.62%	6836	64.22%	0.000
	2~5	1618	22.34%	319	27.38%	837	37.42%	1156	33.98%	2774	26.06%	
	5–10	272	3.76%	79	6.78%	303	13.54%	382	11.23%	654	6.14%	
	> 10	136	1.88%	32	2.75%	212	9.48%	244	7.17%	380	3.57%	
**Detection Mode**	**Passive Case Finding**	4676	64.57%	784	67.30%	1396	62.41%	2180	64.08%	6856	64.41%	0.000
	Spontaneous demand	1580	21.82%	224	19.23%	408	18.24%	632	18.58%	2212	20.78%	
	Dermatology	2033	28.07%	345	29.61%	510	22.80%	855	25.13%	2888	27.13%	
	Other-reported illness	1063	14.68%	215	18.45%	478	21.37%	693	20.37%	1756	16.50%	
	**Active Case Finding**	2472	34.13%	378	32.45%	815	36.43%	1193	35.07%	3665	34.43%	
	Contact examination	963	13.30%	134	11.50%	148	6.62%	282	8.29%	1245	11.70%	
	Focus investigation	246	3.40%	30	2.58%	66	2.95%	96	2.82%	342	3.21%	
	Collective examination	90	1.24%	2	0.17%	6	0.27%	8	0.24%	98	0.92%	
	Clue investigation	1081	14.93%	202	17.34%	572	25.57%	774	22.75%	1855	17.43%	
	General survey	92	1.27%	10	0.86%	23	1.03%	33	0.97%	125	1.17%	
	Other ways	94	1.30%	3	0.26%	26	1.16%	29	0.85%	123	1.16%	
**Contact History**	Absent	2221	30.67%	340	29.18%	736	32.90%	1076	31.63%	3297	30.98%	0.000
	Present	5021	69.33%	825	70.82%	1501	67.10%	2326	68.37%	7347	69.02%	
	Within family	2356	32.53%	376	32.27%	543	24.27%	919	27.01%	3275	30.77%	
	Out of family	2665	36.80%	449	38.54%	958	42.83%	1407	41.36%	4072	38.26%	
**Leprosy Reaction**	Absent	7068	97.60%	1089	93.48%	2189	97.85%	3278	96.36%	10346	97.20%	0.000
	Present	174	2.40%	76	6.52%	48	2.15%	124	3.64%	298	2.80%	
	T1R	68	0.94%	35	3.00%	28	1.25%	63	1.85%	131	1.23%	
	T2R	83	1.15%	31	2.66%	18	0.80%	49	1.44%	132	1.24%	
	Mixed reaction	20	0.28%	11	0.94%	4	0.18%	15	0.44%	35	0.33%	
**Skin Lesion**	0 skin lesion	229	3.16%	44	3.78%	167	7.47%	211	6.20%	440	4.13%	0.000
	1 skin lesion	804	11.10%	107	9.18%	221	9.88%	328	9.64%	1132	10.64%	
	2–4 skin lesions	1984	27.40%	323	27.73%	708	31.65%	1031	30.31%	3015	28.33%	
	≥5 skin lesions	3860	53.30%	659	56.57%	1064	47.56%	1723	50.65%	5583	52.45%	
	Missing data	365	5.04%	32	2.75%	77	3.44%	109	3.20%	474	4.45%	
**Nerve lesion**	0 nerve lesion	942	13.01%	52	4.46%	91	4.07%	143	4.20%	1085	10.19%	0.000
	1 nerve lesion	1441	19.90%	234	20.09%	435	19.45%	669	19.66%	2110	19.82%	
	2 nerve lesions	4716	65.12%	862	73.99%	1675	74.88%	2537	74.57%	7253	68.14%	
	Missing data	143	1.97%	17	1.46%	36	1.61%	53	1.56%	196	1.84%	
**Ridley-Jopling Classification**	LL	1032	14.25%	156	13.39%	222	9.92%	378	11.11%	1410	13.25%	0.000
	BL	3056	42.20%	542	46.52%	713	31.87%	1255	36.89%	4311	40.50%	
	BB	718	9.91%	117	10.04%	210	9.39%	327	9.61%	1045	9.82%	
	BT	1574	21.73%	228	19.57%	747	33.39%	975	28.66%	2549	23.95%	
	TT	717	9.90%	110	9.44%	332	14.84%	442	12.99%	1159	10.89%	
	I	141	1.95%	12	1.03%	10	0.45%	22	0.65%	163	1.53%	
	Missing data	4	0.06%	0	0.00%	3	0.13%	3	0.09%	7	0.07%	
**WHO Classification**	MB	5038	69.57%	849	72.88%	1211	54.14%	2060	60.55%	7098	66.69%	0.000
	PB	2204	30.43%	316	27.12%	1026	45.86%	1342	39.45%	3546	33.31%	
**Diagnosis Duration**	Median (IQR)	13	(7–25)	16	(9–30)	29	(19–56)	24	(12–48)	15	(8–30)	
	Mean±SD (Min-Max)	21.67±30.20	(1–540)	26.06±31.19	(1–288)	49.11±62.52	(1–655)	27.92±40.86	(1–655)	27.92±40.86	(1–655)	

* 1 sample Kolmogorov-Smirnov test

IQR: inter quartile range.

WHO: Operational classification proposed by the World Health Organization.

MB: multibacillary, PB: paucibacillary.

LL:lepromatous lepromatous,BL: borderline lepromatous,BB: midborderline,BT: borderline tuberculoid;TT: tuberculoid tuberculoid; I:inderminate.

T1R: type 1 reaction, T2R: type 2 reaction.

### Risk factors for leprosy disease with G1D

Table 2 summarizes all of the multinomial logistic regression results of risk factors associated with physical impairments graded as G1D and G2D separately when compared with G0D. Han ethnicity, rural occupation, nerve damage, delayed diagnosis, leprosy reaction, zero skin lesions, and Ridley-Jopling classification were associated with a higher risk of G1D.

**Table 2:** Multinominal and ordinal logistic regression of risk factors associated with physical impairment of leprosy disease in Yunnan, China from 1990–2019.

Variables		G1D*		G2D*		Physical disability **	
		OR (95% CI)	P value	OR (95% CI)	P value	OR (95% CI)	P value***
Patient Demographic Characteristics							
[Gender]	Male	0.979(0.851–1.126)	0.764	1.205(1.069–1.358)	0.002***	1.128(1.024–1.243)	0.015***
	Female	1[ref]	.	1[ref]	.	1[ref]	.
[Age]	≥60years	1.159(0.755–1.779)	0.499	2.897(1.895–4.431)	0.000***	2.053(1.498–2.814)	0.000***
	≧15-<59years	0.982(0.676–1.426)	0.942	1.930(1.298–2.869)	0.001***	1.472(1.106–1.960)	0.008***
	<15years	1[ref]	.	1[ref]	.	1[ref]	.
[Ethinics]	Han	1.424(1.249–1.624)	0.000***	1.257(1.127–1.402)	0.000***	1.268(1.159–1.386)	0.000***
	Other ethinics	1[ref]	.	1[ref]	.	1[ref]	.
[Occupation]	Rural	1.332(1.013–1.753)	0.040***	1.418(1.117–1.799)	0.004***	1.364(1.128–1.650)	0.001***
	Urban	1[ref]	.	1[ref]		1[ref]	.
Patient Clinic Characteristics							
[Delay diagnosis]	≥10years	1.722(1.149–2.580)	0.008***	7.469(5.790–9.637)	0.000***	5.652(4.516–7.073)	0.000***
	≧5-<10years	1.938(1.470–2.553)	0.000***	5.752(4.725–7.002)	0.000***	4.399(3.714–5.212)	0.000***
	≧2-<5years	1.284(1.106–1.492)	0.001***	2.848(2.531–3.206)	0.000***	2.275(2.063–2.509)	0.000***
	<2years	1[ref]	.	1[ref]	.	1[ref]	.
[Detection mode]	Self-reported	1.065(0.537–2.113)	0.856	0.857(0.513–1.431)	0.555	0.924(0.599–1.426)	0.721
	Dermatology	1.305(0.661–2.575)	0.443	0.939(0.564–1.563)	0.807	1.043(0.678–1.604)	0.849
	Other-reported	1.549(0.780–3.077)	0.211	1.485(0.889–2.482)	0.131	1.535(0.994–2.370)	0.053
	Contact examination	1.230(0.614–2.462)	0.560	0.662(0.388–1.128)	0.129	0.821(0.526–1.281)	0.385
	Hot spot survey	1.101(0.508–2.386)	0.807	0.972(0.545–1.735)	0.924	0.996(0.661–1.626)	0.988
	Physical examination	0.304(0.064–1.449)	0.135	0.434(0.158–1.190)	0.105	0.388(0.164–0.918)	0.031***
	Clue survey	1.358(0.684–2.698)	0.382	1.418(0.851–2.365)	0.180	1.455(0.944–2.244)	0.089
	General survey	1[ref]	.	1[ref]	.	1[ref]	.
	Active	-		-		-	
	Nagative	1[ref]		1[ref]		1[ref]	.
[Contact history]	Yes	1.135(0.979–1.316)	0.093	1.028(0.912–1.158)	0.653	1.043(0.946–1.151)	0.397
	No	1[ref]	.	1[ref]	.	1[ref]	.
[Skin lesion]	0 lesion	1.666(1.158–2.395)	0.006***	2.037(1.552–2.675)	0.000***	1.916(1.522–2.413)	0.000***
	1 lesion	1.105(0.843–1.450)	0.470	0.830(0.667–1.032)	0.094	0.865(0.722–1.035)	0.114
	2–4 lesion	1.107(0.943–1.300)	0.215	1.000(0.875–1.142)	0.996	1.028(0.922–1.147)	0.619
	≥5 lesion	1[ref]	.	1[ref]	.	1[ref]	.
[Nerve damage]	2 nerve	3.130(2.314–4.233)	0.000***	3.686(2.869–4.736)	0.000***	3.474(2.843–4.244)	0.000***
	1 nerve	2.849(2.063–3.934)	0.000***	2.320(1.775–3.031)	0.000***	2.428(1.959–3.008)	0.000***
	0 nerve	1[ref]	.	1[ref]	.	1[ref]	.
[Leprosy reaction]	Yes	3.196(2.369–4.312)	0.000***	1.115(0.771–1.612)	0.563	1.528(1.195–1.952)	0.001***
	No	1[ref]	.	1[ref]	.	1[ref]	.
	T1R	3.354(2.166–5.193)	0.000***	1.259(0.758–2.091)	0.374	1.605(1.131–2.277)	0.008***
	T2R	2.928(1.865–4.598)	0.000***	1.190(0.672–2.108)	0.551	1.565(1.075–2.280)	0.020***
	Mixed	4.074(1.744–9.522)	0.001***	1.283(0.411–4.002)	0.668	1.869(0.926–3.774)	0.081
	No	1[ref]		1[ref]		1[ref]	
[R-J classification]	I	0.764(0.355–1.643)	0.490	0.348(0.159–0.761)	0.008***	0.482(0.273–0.853)	0.012***
	TT	1.177(0.853–1.624)	0.794	1.753(1.163–2.643)	0.007***	1.479(1.052–2.079)	0.024***
	BT	1.055(0.827–1.346	0.895	1.694(1.189–2.415)	0.004***	1.438(1.075–1.923)	0.014***
	BB	1.121(0.854–1.472)	0.411	1.707(1.353–2.153)	0.000***	1.522(1.261–1.838)	0.000***
	BL	1.259(1.030–1.540)	0.025***	1.211(1.010–1.452)	0.039***	1.239(1.072–1.431)	0.004***
	LL	1[ref]	.	1[ref]	.	1[ref]	.
[WHO classification]	MB	1[ref]		1[ref]		1[ref]	.
	PB	1.093(0.724–1.651)	0.673	1.925(1.389–2.667)	0.000***	1.759(1.341–2.307)	0.000***

G0D, grade 0 disability; G1D, grade 1 disability; G2D, grade 2 disability; OR, odds ratio; CI, confidence interval.

*Multinominal logistic regression. The reference catogory is G0D.

**Ordinal logistic regression. dependent variable: disability.

***P <0.05.T1R: type 1 reaction, T2R: type 2 reaction.

Regarding ethnicity, Han ethnicity increased the risk of G1D compared with minority ethnicities (OR: 1.424; 95% CI: 1.249–1.624; P = 0.000). Regarding occupation, working in rural areas increased risk of G1D compared with working in urban areas (OR: 1.332; 95% CI: 1.013–1.753; P = 0.040). Regarding nerve damage, one (OR: 2.849; 95% CI: 2.063–3.934; P = 0.000) or 2 (OR: 3.130; 95% CI: 2.314–4.233; P = 0.000) damaged nerves increased the risk of G1D compared with no nerve damage. Regarding leprosy reactions, patients with leprosy reactions had an increased risk of G1D compared with patients without leprosy reactions (OR: 3.196; 95% CI: 2.369–4.312; P = 0.000). Furthermore, type 1 leprosy reactions (T1R) (OR: 3.354; 95% CI: 2.166–5.193; P = 0.000), T2R (OR: 2.928; 95% CI: 1.865–4.598; P = 0.000), and mixed leprosy reactions (OR: 4.074; 95% CI: 1.744–9.522; P = 0.001) increased the risk of G1D compared with no leprosy reaction. Regarding the diagnosis duration, 2–5 years (OR: 1.284; 95% CI: 1.106–1.492; P = 0.001), 5–10 years (OR: 1.938; 95% CI: 1.470–2.553; P = 0.000), and over 10 years (OR: 1.722; 95% CI: 1.149–2.580; P = 0.008) increased the risk of G1D compared with less than 2 years. Having zero skin lesions increased the risk of G1D compared with having more than 5 skin lesions (OR: 1.666; 95% CI: 1.158–2.395; P = 0.006). Regarding the Ridley-Jopling classification, the BL form increased the risk of G1D compared with the LL form (OR: 1.259; 95% CI: 1.030–1.540; P = 0.025). However, there were no interactions of sex, age, detection mode, contact history, or WHO operational classification with the risk of developing G1D (Table 2).

### Risk factors for leprosy disease with G2D

Advanced age, rural occupation, Han ethnicity, male sex, delayed diagnosis, nerve damage, zero skin lesions, WHO and Ridley-Jopling classifications, and leprosy reactions were also associated with a higher risk of G2D analyzed by multinomial logistic regression (Table 2).

Regarding age, being 15–59 years old (OR: 1.930; 95% CI: 1.298–2.869; P = 0.001) and over 60 years old (OR: 2.897; 95% CI: 1.895–4.431; P = 0.000) increased the risk of G2D compared with being under 15 years old. Regarding occupation, working in rural areas increased risk of G2D compared with working in urban areas (OR: 1.418; 95% CI: 1.117–1.799; P = 0.004). Regarding ethnicity, Han ethnicity increased the risk of G2D compared with minority ethnicities (OR: 1.257; 95% CI: 1.127–1.402; P = 0.000). Regarding sex, males had an increased risk of G2D compared with females (OR: 1.205; 95% CI: 1.069–1.358; P = 0.002).

Regarding diagnosis duration, 2–5 years (OR: 2.848; 95% CI: 2.531–3.206; P = 0.000), 5–10 years (OR: 5.752; 95% CI: 4.725–7.002; P = 0.000), and over 10 years (OR: 7.469; 95% CI: 5.790–9.637; P = 0.000) increased the risk of G2D compared with less than 2 years. Regarding nerve damage, damage to one (OR: 2.320; 95% CI: 1.775–3.031; P = 0.000) or 2 (OR: 3.686; 95% CI: 2.869–4.736; P = 0.000) nerves increased the risk of G2D compared with no nerve damage. Having zero skin lesions increased the risk of G2D compared with having more than 5 skin lesions (OR: 2.037; 95% CI: 1.552–2.675; P = 0.000). Regarding the WHO classification, the PB form (OR: 1.925; 95% CI: 1.389–2.667; P = 0.000) increased the risk of G2D compared with the MB form (Table 2). Regarding the clinical classification, TT (OR: 1.753; 95% CI: 1.163–2.634; P = 0.007), BT (OR: 1.694; 95% CI: 1.189–2.415; P = 0.004), BB (OR: 1.707; 95% CI: 1.353–2.153; P = 0.000), and BL (OR: 1.211; 95% CI: 1.010–1.452; P = 0.039) increased the risk of G2D compared with LL (Table 2). The I form (OR: 0.348; 95% CI: 0.159–0.761; P = 0.008) decreased the risk of G2D compared with the LL form (Table 2). Regarding leprosy reactions, patients with leprosy reactions had an increased risk of G1D compared with patients without leprosy reactions, but there was no impact on G2D (P = 0.563) (Table 2). Similar to findings with G1D, no association of detection mode or contact history and risk of G2D was found (Table 2).

### Risk factors for physical disabilities and deformity associated with leprosy disease

Table 2 also summarizes the results of risk factors associated with physical disabilities (combined G1D and G2D) by ordinal logistic regression. Advanced age, rural occupation, Han ethnicity, male sex, delayed diagnosis, nerve damage, zero skin lesions, WHO and Ridley-Jopling classifications, and leprosy reactions were associated with a higher risk of physical disability associated with leprosy disease.

Regarding age, being 15–59 years old (OR: 1.472; 95% CI: 1.106–1.960; P = 0.008) and over 60 years old (OR: 2.053; 95% CI: 1.498–2.814; P = 0.000) had an increased risk of physical disability compared with those being under 15 years old. Regarding occupation, working in rural increased risk of G2D compared with working in urban areas (OR: 1.364; 95% CI: 1.128–1.650; P = 0.001). Regarding ethnicity, Han ethnicity increased the risk of physical disability compared with minority ethnicities (OR: 1.268; 95% CI: 1.159–1.386; P = 0.000). Regarding sex, males were at higher risk of physical disability due to leprosy disease than females (OR: 1.128; 95% CI: 1.1.024–1.243; P = 0.015).

Regarding diagnosis duration, 2–5 years (OR: 2.275; 95% CI: 2.063–2.509; P = 0.000), 5–10 years (OR: 4.399; 95% CI: 3.714–5.212; P = 0.000), and over 10 years (OR: 5.652; 95% CI: 4.516–7.073; P = 0.000) increased the risk of disability compared with less than 2 years. Regarding nerve damage, damage to one nerve (OR: 2.428; 95% CI: 1.959–3.008; P = 0.000) and 2 nerves (OR: 3.474; 95% CI: 2.843–4.244; P = 0.000) increased the risk of G2D compared with no nerve damage. Regarding skin lesions, those without skin lesions had an increased risk of disability compared with those with more than 5 skin lesions (OR: 1.916; 95% CI: 1.522–2.413, P = 0.000). Regarding the WHO classification, the PB form (OR: 1.759; 95% CI: 1.341–2.307; P = 0.000) increased the risk of physical disability compared with the MB form (Table 2). Regarding clinical classifications, TT (OR: 1.479; 95% CI: 1.052–2.079; P = 0.024), BT (OR: 1.438; 95% CI: 1.075–1.923; P = 0.014), BB (OR: 1.522; 95% CI: 1.261–1.838; P = 0.000), and BL (OR: 1.239; 95% CI: 1.072–1.431; P = 0.004) increased the risk of physical disability compared with LL (Table 2). Regarding leprosy reactions, leprosy reactions increased the risk of disability compared with the absence of leprosy reactions (OR: 1.528; 95% CI: 1.195–1.952; P = 0.001). Furthermore, T1R (OR: 1.605; 95% CI: 1.131–2.277; P = 0.008) and T2R (OR: 1.565; 95% CI: 1.075–2.280; P = 0.020) increased the risk of physical disability compared with the absence of leprosy reactions. Among the detection modes, those receiving a physical examination (OR: 0.398; 95% CI: 0.168–0.941, P = 0.036) demonstrated a lower risk of developing disability than those identified through a general surveys. No associations between contact history and physical disabilities associated with leprosy were detected.

## Discussion

We report 3402 leprosy patients with G1D and G2D and confirmed physical disabilities due to leprosy disease during 1990–2019 in Yunnan, China. G1D and G2D accounted for 10.95% and 21.02% of the total sample of patients, respectively. Advanced age, rural occupation, Han ethnicity, male sex, delayed diagnosis, nerve damage, WHO and Ridley-Jopling classifications, zero skin lesions, and leprosy reactions were associated with a high risk of developing physical disability associated with leprosy disease.

Advanced age at diagnosis has been previously reported as a risk predictor for developing a physical disability associated with leprosy disease [14]. Age is known to be related to disease duration and diagnosis delay [15,16]. This study quantified the association of advanced age and physical disability since the study was conducted in Brazil [14]. Male patients were not associated with the risk of G1D but were 1.205- and 1.128-times more likely than female patients to have G2D and overall physical disabilities, respectively. Similar results were observed in studies conducted in China [17], Burkina Faso [18], Turkey [19], Zimbabwe [20], Bangladesh [21], India [22], Brazil [1,23–28], Indonesia [9], and Nigeria [29]. Male patients were more likely to have physical disabilities than female patients [30], which may be due to social behaviors [31]. Our study also indicated that a rural occupation was associated with a risk of physical disability. which may be due to socioeconomic status, such as undernutrition, and be associated with a reduced expenditure on food, possibly brought on by increased unemployment and a loss of income [32,33].

In another study, an active detection mode was demonstrated to be associated with a higher risk of physical disability than a passive detection mode [14]. This finding was reported in the Brazilian population, and the diagnosis of leprosy using community surveys (e.g., schools, nurseries, small villages) increased the detection of G2D. This may reflect the better training of the examiners, with a defined focus on verifying leprosy complications [14]. Active and passive detection modes should not be compared competitively but rather need to be interpreted as mutually enhancing for the effective early detection of leprosy, which in turn can reduce G2D [34]. However, in this study, statistically significant differences were not found between the active and passive detection modes with regard to the presence of disabilities.

Early identification combined with the proper treatment of leprosy reactions can be an effective strategy to prevent disability in leprosy patients [23]. In contrast, a longer diagnosis duration was 2–5 times more likely to develop physical disabilities than a diagnosis within 2 years in this study. In our analysis, the variable of longer diagnosis duration presented the highest odds for developing physical disability. Similar trends were also found with G2D and G1D.

Nerve damage and leprosy reactions have been reported as risk factors for developing physical disability in previous studies [35–38]. The multinomial and ordinal logistic regression analysis findings support previous studies showing that the number of affected nerves upon diagnosis was associated with G1D, G2D, and combined physical disabilities (G1D and G2D). The presence of a leprosy reaction was associated with G1D and combined physical disabilities.

Regarding the WHO and Ridley-Jopling classifications, the PB and Ridley-Jopling classifications tended toward the TT form being associated with G2D and combined physical disabilities. The results showed that zero skin lesions was associated with a greater risk to develop G1D, G2D, and combined physical disabilities in Yunnan, China, which was different from findings in other studies [30]. Most studies have demonstrated that male sex, multibacillary leprosy, leprosy reactions, and lepromatous presentation were associated with the presence of physical disabilities associated with leprosy [30], but only a few studies showed the relationship between PB and physical disabilities [39]. As the epidemiology of the disease varies greatly worldwide, a certain population may present specific risk factors for disabilities, and there may be factors common to all populations. The different statistical analysis methods could be another probable cause of discrepancies among studies. In a previous study, only one factor based on prevalence was included in the model at one time. In this study, various factors were included, and logistic regression showed which of the various factors being assessed had the strongest association with an outcome and provided a measure of the magnitude of the potential influence. Logistic regression also has the ability to "adjust" for confounding factors, i.e., factors that are associated with both other predictor variables and the outcome, such that the measure of the influence of the predictor of interest is not distorted by the effect of the confounder [40].

This study should be interpreted within the context of its limitations. The main limitation of this study is that it was a retrospective observational study; therefore, the results relied on the accuracy and completeness of patient records. The LEPMIS was established by the National Center for Leprosy Control in 2010, and the data have since become more accurate and integrated. During the past several decades, the definitions for the WHO classifications of MB and PB have been continually adjusted. These classifications may have influenced the WHO operational classification indicators used in this study.

## Conclusion

In conclusion, the most important risk factors for physical disability at diagnosis, in decreasing order of importance, were delayed diagnosis, nerve damage, paucibacillary leprosy, Ridley-Jopling classification as tuberculoid, advanced age, zero skin lesions, leprosy reactions, rural occupation, Han ethnicity, and male sex. The identification of risk factors associated with physical disability in this study could be helpful for disability prevention in the endemic population in Yunnan, China. Early detection and proper therapy with multidrug therapy (MDT) are necessary to reduce the burden of disability and deformity associated with leprosy disease.
